# Diagnostic value of magnetic resonance imaging ectopic posterior pituitary hyperintense signal in pituitary macroadenoma

**DOI:** 10.3389/fonc.2022.971730

**Published:** 2022-11-11

**Authors:** Yi-Lin Luo, Fang Gu, Hai-Qing Fan, Jin-Hui Du, Yue Yu, Li-Kun Liu, Xin Liao

**Affiliations:** ^1^ Department of Medical Imaging, The Second People’s Hospital of Guiyang, Guiyang, China; ^2^ Department of Medical Imaging, The Affiliated Hospital of Guizhou Medical University, Guiyang, China

**Keywords:** pituitary macroadenoma, ectopic posterior pituitary hyperintense signal, magnetic resonance imaging, diagnosis, sellar lesion

## Abstract

**Objective:**

When the lesions in the sellar region are large, they can involve both the inside and outside the sella, which brings challenges to the differential diagnosis of pituitary macroadenoma and lesions other than macroadenoma. Therefore, this study explored the diagnostic value of an ectopic posterior pituitary hyperintense signal (EPPHS) in pituitary macroadenoma and its possible causes.

**Methods:**

The clinical and imaging data of 131 patients with sellar tumors or tumor-like lesions involving both intrasellar and extrasellar regions in the Affiliated Hospital of Guizhou Medical University from February 2011 to December 2021 were analyzed retrospectively. The diagnostic value of EPPHS in pituitary macroadenoma was analyzed. The differences in clinical and imaging indexes between the EPPHS-positive group and the EPPHS-negative group were compared.

**Results:**

These 131 cases of sellar tumors or tumor-like lesions involving both intrasellar and extrasellar regions included 91 cases of pituitary macroadenoma and 40 cases of lesions other than macroadenoma. The receiver operator characteristic (ROC) curve analysis suggested that EPPHS had a diagnostic value in diagnosing pituitary macroadenoma [area under the curve (AUC) = 0.857, *P* = 0.0001]. Compared with the EPPHS negative group, the median prolactin level in the EPPHS positive group was significantly higher (*P* < 0.05). Through ROC curve analysis, prolactin value was found to be of diagnostic value for EPPHS (AUC = 0.612, *P* = 0.0312).

**Conclusion:**

In sellar tumors or tumor-like lesions involving both intrasellar and extrasellar regions, the appearance of EPPHS is helpful in the diagnosis of pituitary macroadenoma. The formation of EPPHS may be related to injuries to the pituitary stalk.

## Introduction

The sellar region is a complex anatomical region. This relatively narrow space contains multiple important tissue structures, so a variety of diseases occur in this area; among these diseases, pituitary adenoma is the most common lesion in the sellar region (1). It is essential to correctly identify these different sellar diseases before an operation because the best treatment strategies for various diseases differ, and a wrong preoperative diagnosis can cause unnecessary harm to patients (2–7). However, when the lesions in the sellar region are large and involve both intrasellar and extrasellar regions, the normal pituitary is often difficult to identify. At this time, it becomes difficult to distinguish between pituitary macroadenoma and lesions other than macroadenoma. Ectopic posterior pituitary hyperintense signal (EPPHS) has a high incidence in pituitary macroadenomas. However, whether there is a difference in the incidence of EPPHS between pituitary macroadenoma and lesions other than macroadenoma in the sellar region has not yet been studied and reported. This study compared and analyzed the incidence of EPPHS in different sellar lesions and found that it can be used as a valuable diagnostic sign of pituitary macroadenoma. The possible causes for this are also discussed in this study.

## Data and methods

### Clinical data

The clinical and imaging data of patients with sellar tumors or tumor-like lesions involving both the intrasellar and extrasellar regions in the Affiliated Hospital of Guizhou Medical University from February 2011 to December 2021 were analyzed retrospectively. Inclusion criteria: (1) At least one non-enhanced magnetic resonance imaging (MRI) of the sellar region was performed before the operation; (2) Patients with pathological results after the operation. Exclusion criteria: (1) There were widespread hyperintense signals in the lesions on the T1 weighted image (T_1_WI), which affected the reading of the anatomical structures; (2) Patients with a history of radiotherapy and chemotherapy before the operation. Finally, 131 patients were included in the study. The pituitary hormone levels of the patients were collected, including the thyroid-stimulating hormone (TSH), adrenocorticotrophic hormone (ACTH), luteinizing hormone (LH), follicle-stimulating hormone (FSH), growth hormone (GH), and prolactin (PRL).

### Detection methods

A Philips Achieva 3.0 TX-Series superconducting MRI system (USA) was used in this study. Sagittal T_1_WI images were taken using spin echo (SE). The time of repetition (TR)/time of echo (TE) was 400/12 ms, the acquisition matrix was 156 × 125, and the slice thickness was 3 mm. Sagittal T2 weighted imaging (T_2_WI) images were taken using fast spin echo (FSE). The TR/TE was 3,500/100 ms, the acquisition matrix was 168 × 133, and the slice thickness was 3 mm. Coronal T_1_WI images were taken using SE, the TR/TE was 500/20 ms, the acquisition matrix was 136 × 109, and the slice thickness was 2.5 mm. Coronal T_2_WI images were taken using FSE, the TR/TE was 3,000/90 ms, the acquisition matrix was 168 × 126, and the slice thickness was 2.5 mm. Gadolinium diethylenetriaminepentaacetic acid was bolus injected by high-pressure syringe through the elbow vein, the dose was 0.2 mL/kg, and the injection rate was 2.5–3.0 mL/s. Coronal and sagittal T_1_WI scans were performed after the injection of the contrast medium. After scanning, all images were transmitted to the picture archiving and communication system (PACS).

### Image analysis

Two experienced central nervous system radiologists evaluated these imaging data at PACS workstation and reached a consensus. According to the imaging data, the general imaging characteristics of the sellar lesions were analyzed, including the size of the lesion, the texture of the lesion (solid, cystic-solid, cystic), and the enhancement characteristics (with or without enhancement). The imaging manifestations of the posterior pituitary in different sellar diseases were observed on T_1_WI. When the posterior pituitary hyperintense signal was located within the sella turcica, it was recorded as a normal posterior pituitary hyperintense signal. When the hyperintense signal of the posterior pituitary was located outside the sella, it was recorded as EPPHS, and its specific location and morphology were recorded. When there was no hyperintense signal of the posterior pituitary on the coronal and sagittal T_1_WI images, it was recorded that the hyperintense signal of the posterior pituitary undetected.

### Statistical analysis

All the data in this study were built and entered in Excel, and statistical analysis was performed using SPSS 22.0 software. Count data were expressed as n (%) and compared between the two groups using Chi-square tests. Normally distributed measurement data were expressed as the mean ± standard deviation (M ± SD) and compared between two groups using independent sample t-tests. Non-normally distributed measurement data were compared using a nonparametric Mann–Whitney U test and a Kolmogorov–Smirnov test. The receiver operating characteristic (ROC) curve was used to determine the diagnostic value of the indicators, and *P* < 0.05 was considered statistically significant.

## Results

### Patient characteristics

A total of 131 patients were included in this research, including 59 men and 72 women. The age of these patients ranged from 5 to 76, with an average of 48.2 ± 15.6 years old. The diagnoses of these 131 patients included pituitary macroadenoma (n = 91), meningioma (n = 14), Rathke cleft cyst (n = 9), craniopharyngioma (n = 8), germ cell tumor (n = 4), metastasis (n = 2), glioma (n = 1), pituitary abscess (n = 1), and pituitary cryptococcal granuloma (n = 1).

### Imaging manifestations of different sellar diseases

**Figure 1:**
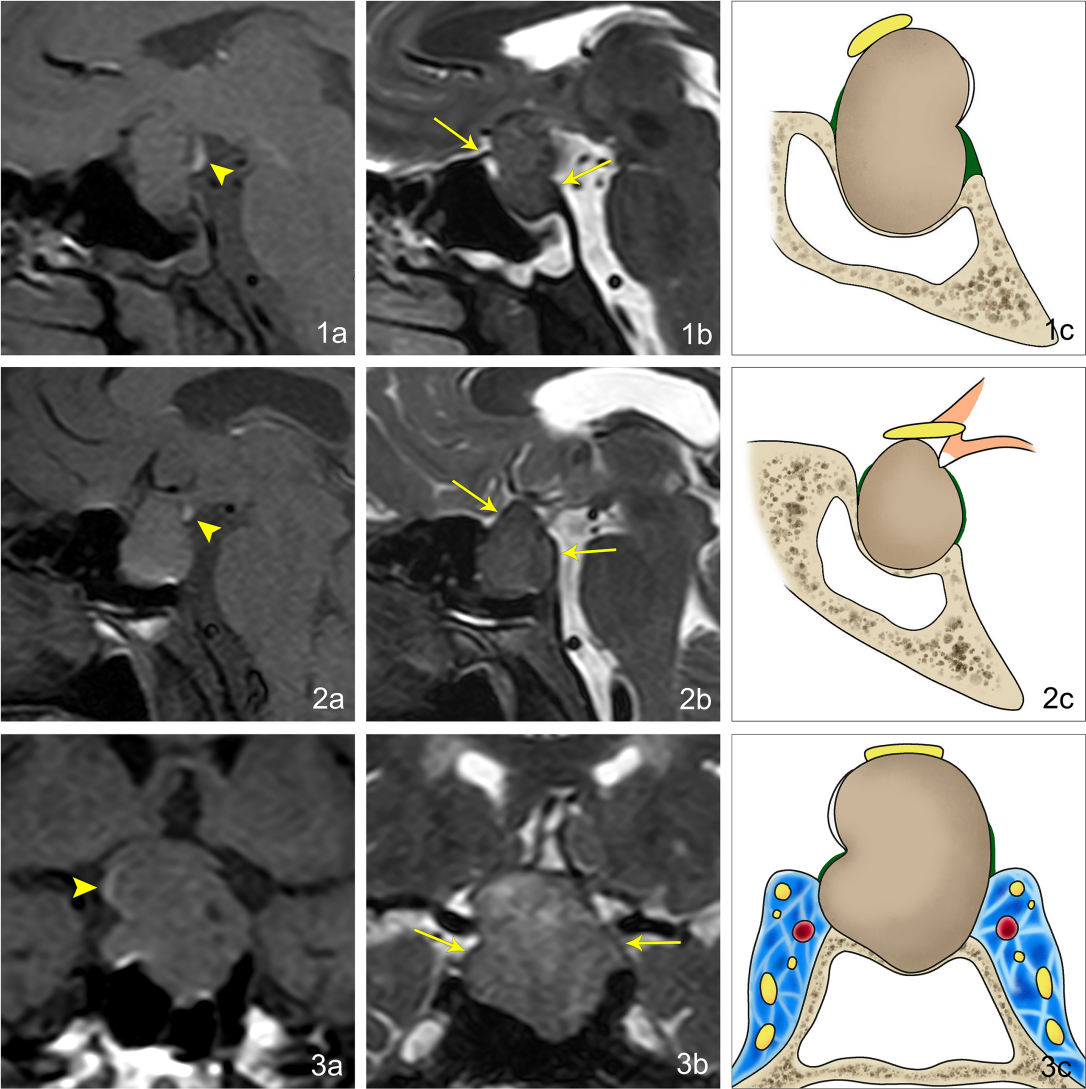
Imaging manifestations of EPPHS at different positions in pituitary macroadenoma. **(1A)** Sagittal T_1_WI reveals that EPPHS is arc-shaped and hyperintense, which is located at the posterior upper edge of pituitary macroadenoma (arrowhead). **(1B)** Sagittal T_2_WI reveals that the residual sellar septum at the anterior and posterior edges of pituitary macroadenoma is a strip-shaped hypointense signal (arrow). **(1C)** Pattern diagram reveals that the arc-shaped white structure is EPPHS, the brown structure is the pituitary macroadenoma, the yellow structure is the optic nerve, the green structure is the sellar septum, and the yellow-gray structure is the sella turcica. **(2A)** Sagittal T_1_WI reveals that EPPHS is nodular and hyperintense, which is located at the distal end of the pituitary stalk (arrowhead). **(2B)** Sagittal T_2_WI reveals that the residual sellar septum at the anterior and posterior edges of the pituitary macroadenoma is the strip-shaped hypointense signal (arrow). **(2C)** Pattern diagram reveals the arc-shaped white structure is EPPHS, the orange structure is the hypothalamus and pituitary stalk, the brown structure is the pituitary macroadenoma, the yellow structure is the optic nerve, the green structure is the sellar septum, and the yellow-gray structure is the sella turcica. **(3A)** Sagittal T_1_WI reveals that EPPHS is arc-shaped and hyperintense, which is located at the right margin of pituitary (arrowhead). **(3B)** Sagittal T_2_WI reveals that the residual sellar septum at the left and right edges of the pituitary macroadenoma is a strip-shaped hypointense signal (arrow). **(3C)** Pattern diagram reveals the arc-shaped white structure is EPPHS, the brown structure is the pituitary macroadenoma, the yellow structure is the optic nerve, the green structure is the sellar septum, the yellow-gray structure is the sella turcica, and the blue area is the cavernous sinus.

**Figure 2:**
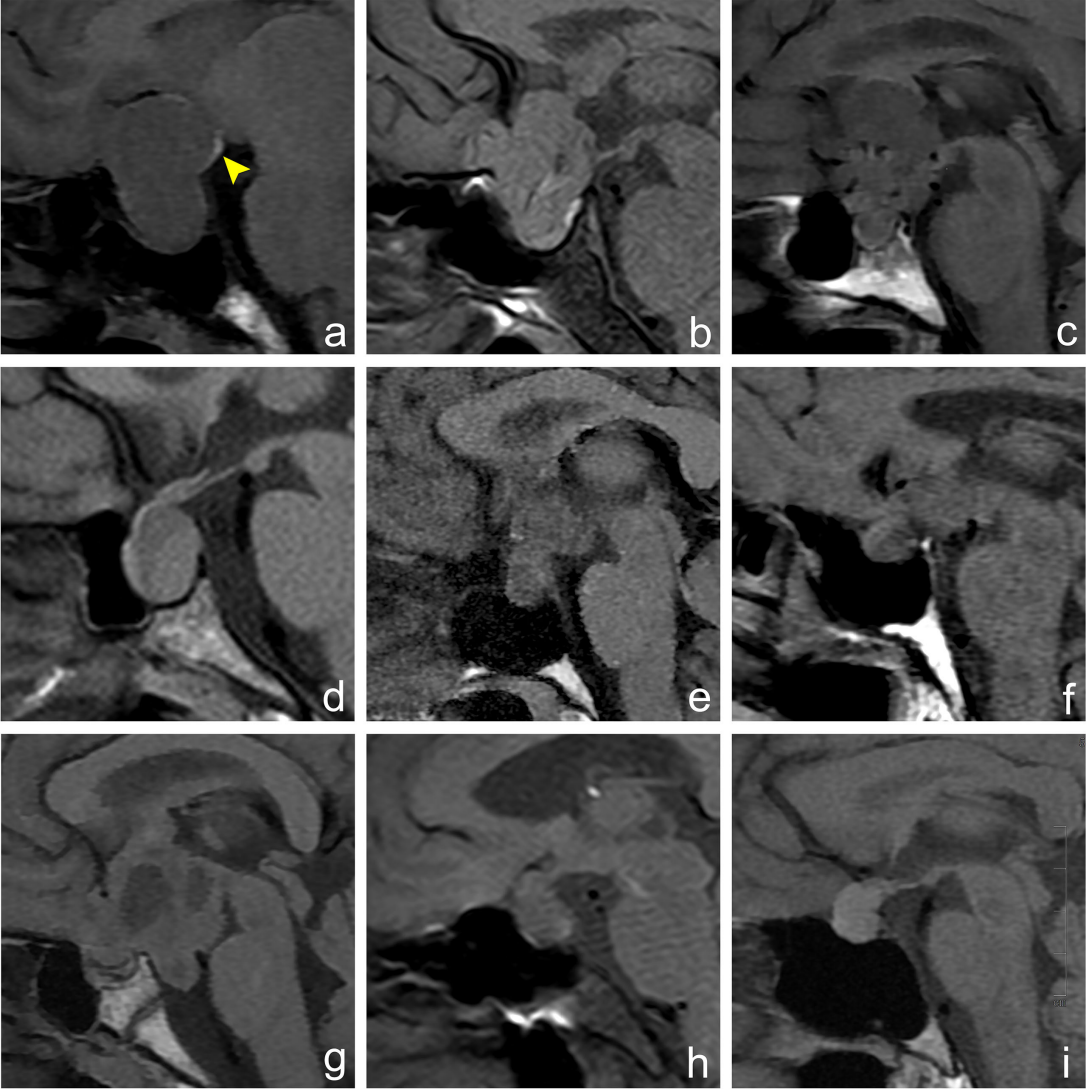
Imaging manifestations of hyperintense signal in posterior pituitary in different sellar diseases. Figures **(A–I)** show an MRI sagittal T_1_WI non-enhanced scan of different lesions in the sellar region. **(A)** A 30-year-old female, pituitary macroadenoma, ectopic hyperintense signal in the posterior pituitary, which is located at the posterior upper edge of the tumor (arrowhead). **(B)** A 64-year-old male, meningioma, normal hyperintense signal in the posterior pituitary, which is located behind the intrasellar adenohypophysis. **(C)** A 39-year-old male, craniopharyngioma, hyperintense signal in the posterior pituitary disappears. **(D)** A 54-year-old female, Rathke cleft cyst, hyperintense signal in the posterior pituitary disappears. **(E)** A 35-year-old female, germ cell tumor, hyperintense signal in the posterior pituitary disappears. **(F)** A 60-year-old male, metastatic tumor, hyperintense signal in the posterior pituitary disappears. **(G)** A 14-year-old female, glioma, hyperintense signal in the posterior pituitary disappears. **(H)** A 56-year-old female, cryptococcal granuloma of pituitary, hyperintense signal in the posterior pituitary disappears. **(I)** A 40-year-old female, abscess of the pituitary, hyperintense signal in the posterior pituitary disappears.

In 91 cases of pituitary macroadenoma, the tumor height was 15.0–60.0 mm. In 83 of the patients, it was ≥20.0 mm, with an average height of about 30.9 mm. In 40 cases of lesions other than macroadenoma in the sellar region, the tumor height was 10.0–65.0 mm. In 26 of the patients, it was ≥20.0 mm, with an average height of about 25.2 mm (**Table 1**). In 65 of the 91 cases of pituitary macroadenomas, EPPHS was found. On T_1_WI, it showed strip or nodular hyperintense signals, which were mainly located at the posterior edge (n = 36) and lateral edge (n = 12) of the tumor and the distal end of the pituitary stalk (n = 17) (**Figure 1**). No EPPHS was found in the other 40 cases other than macroadenoma in the sellar region (**Figure 2**).

**Table 1:** The imaging features of different sella area diseases.

Sellar lesion	Lesion texture			Hyperintense signal in posterior pituitary			Enhancement
	Solid	Cystic-solid	Cystic	Normal	Ectopic	Undetected	
Pituitary macroadenoma (n=91)	76.9% (70/91)	23.1% (21/91)	0% (0/91)	5.5% (5/91)	71.4% (65/91)	23.1% (21/91)	+
Meningioma (n=14)	100% (14/14)	0% (0/14)	0% (0/14)	85.7% (12/14)	0% (0/14)	14.3% (2/14)	+
Rathke cleft cyst (n=9)	0% (0/9)	0% (0/9)	100% (9/9)	44.4% (4/9)	0% (0/9)	55.6% (5/9)	−
Craniopharyngioma (n=8)	0% (0/8)	62.5% (5/8)	37.5% (3/8)	25.0% (2/8)	0% (0/8)	75.0% (6/8)	+
Germ cell tumor (n=4)	50.0% (2/4)	50.0% (2/4)	0% (0/4)	0% (0/4)	0% (0/4)	100% (4/4)	+
Metastatic tumor (n=2)	100% (2/2)	0% (0/2)	0% (0/2)	0% (0/2)	0% (0/2)	100% (2/2)	+
Glioma (n=1)	0% (0/1)	100% (1/1)	0% (0/1)	0% (0/1)	0% (0/1)	100% (1/1)	+
Pituitary abscess (n=1)	0% (0/1)	0% (0/1)	100% (1/1)	0% (0/1)	0% (0/1)	100% (1/1)	−
Cryptococcal granuloma of pituitary (n=1)	100% (1/1)	0% (0/1)	0% (0/1)	0% (0/1)	0% (0/1)	100% (1/1)	+

“+” indicates enhancement, “-” indicates no enhancement.

### Comparison of the EPPHS detection rate between pituitary macroadenoma and lesions other than macroadenoma

**Figure 3:**
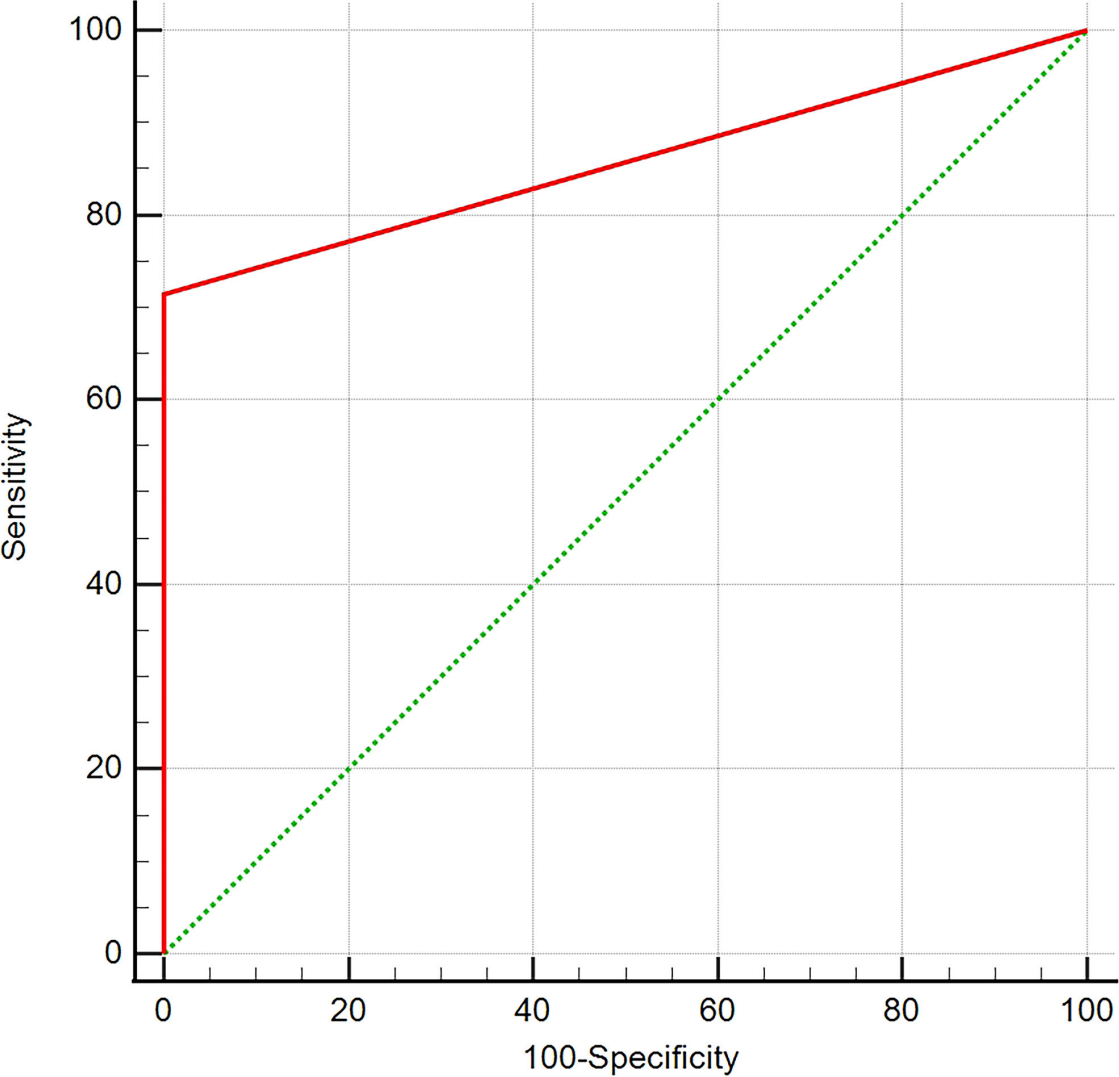
ROC curve analysis. The analysis of ROC curve reveals that EPPHS has diagnostic value in diagnosis of pituitary macroadenoma.

On T_1_WI, the detection rate of EPPHS was 71.4% in pituitary macroadenomas (65/91) and 0% (0/40) in lesions other than macroadenoma. The ROC curve analysis suggested that EPPHS had a diagnostic value in the diagnosis of pituitary macroadenoma (area under the curve [AUC] = 0.857, *P* = 0.0001, **Figure 3**).

### Comparison of various indexes between the EPPHS-positive group and EPPHS-negative group

**Figure 4:**
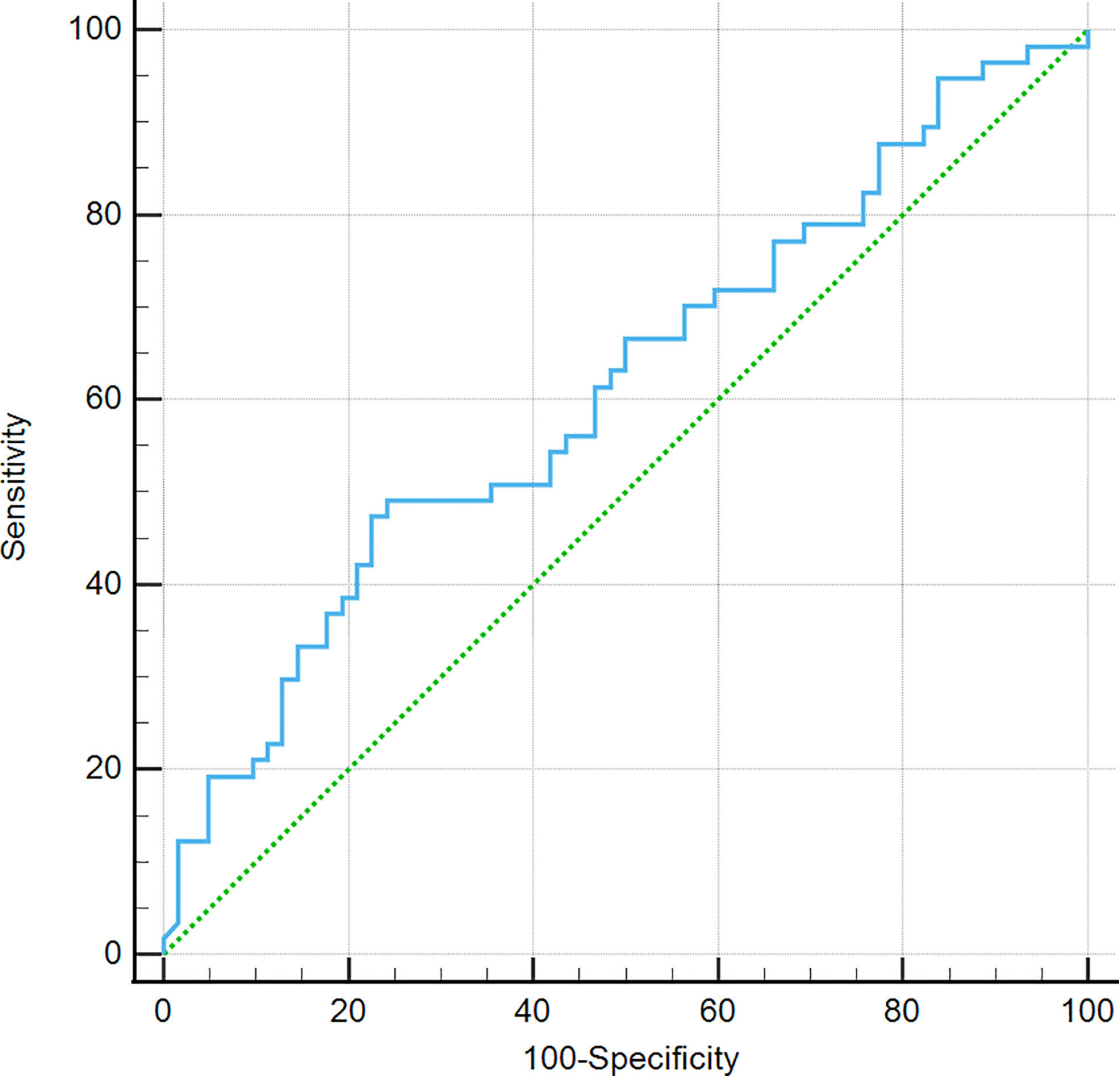
ROC curve analysis. It was found that prolactin value is of diagnostic value for EPPHS through the analysis of work curve (ROC curve).

There were no significant differences in age, gender, right-to-left diameter, anterior–posterior diameter, height, TSH, ACTH, FSH, LH, and GH between the EPPHS-positive group and the EPPHS-negative group (*P* > 0.05). Only the median PRL level in the EPPHS positive group was significantly higher than in the EPPHS negative group (*P* < 0.05, **Table 2**). The ROC curve analysis showed that the prolactin value had diagnostic value for EPPHS (AUC = 0.612, *P* = 0.0312, **Figure 4**).

**Table 2:** Comparison of indicators of EPPHS negative and positive groups.

	EPPHS positive group	EPPHS negative group	*P* value
Age (mean ± SD)	50.00 ± 13.86	46.34 ± 17.00	0.181
Gender	Male (32, 50.8%) Female (33, 49.2%)	Male (27, 40.9%) Female (39, 59.1%)	0.338
Right-to-left diameter (mm) (M, P25, P75)	21 (20.81, 24.79)	22.5 (20.64, 24.69)	0.120
Anterior-posterior diameter (mm) (M, P25, P75)	26 (24.06, 27.73)	23 (21.33, 25.88)	0.067
Height (mm) (M, P25, P75)	29 (28.22, 32.94)	27 (25.20, 30.29)	0.094
TSH mIU/L (M, P25, P75)	2.65 (2.22, 3.43)	1.75 (1.75, 3.23)	0.062
ACTH pg/mL (M, P25, P75)	24.55 (20.90, 27.53)	23.55 (21.56, 28.19)	0.809
PRL mIU/L (M, P25, P75)	568.95 (487.16, 1154.54)	339.21 (342.43, 616.83)	0.017
FSH IU/L (M, P25, P75)	7.00 (7.10, 14.22)	5.72 (9.85, 18.69)	0.726
LH IU/L (M, P25, P75)	2.54 (2.68, 4.08)	4.08 (4.97, 14.26)	0.093
GH ug/lL (M, P25, P75)	0.22 (0.79, 4.22)	0.24 (0.24, 3.97)	0.915

## Discussion

The normal posterior pituitary is located in the sella turcica and shows hyperintense signals on T_1_WI. These hyperintense signals are related to the storage of antidiuretic hormones in neuroendocrine granules (8). In some congenital or acquired lesions, this hyperintense signal can be ectopic to other extrasellar locations, called EPPHS. In congenital lesions, EPPHS is mainly found in pituitary stalk blocking syndrome, usually near the median eminence, and it is often accompanied by the absence of pituitary stalk and adenohypophyseal dysplasia (9). Acquired EPPHS is rare and is mainly reported in pituitary macroadenomas. In pituitary macroadenomas with a height greater than 20 mm, its incidence is very high (10). In this study, of 91 cases of pituitary macroadenomas, 91.2% of pituitary macroadenomas were ≥2 cm in height, and 71.4% of them had EPPHS. No EPPHS was found in the other 40 cases of tumors or tumor-like lesions involving both the inside and outside of the sella. The ROC curve analysis suggested that EPPHS had diagnostic value in the diagnosis of pituitary macroadenoma.

There are rarely investigations on the formation mechanism of EPPHS in pituitary macroadenoma. By observing and analyzing the morphology of pituitary macroadenoma, Saeki believed that the formation of EPPHS in pituitary macroadenomas was related to the compression of the transverse septum on the hypothalamic neurohypophyseal axis (11). The present study revealed that the median PRL level was significantly higher in the EPPHS-positive group than in the EPPHS-negative group. It also suggested that the pituitary stalk may be more compressed in the EPPHS-positive group (12, 13). Saeki believed that it is precisely this compression of the pituitary stalk that causes neuroendocrine particles to accumulate locally at the distal end of the pituitary stalk, which is manifested as EPPHS on T_1_WI non-enhanced images (11). However, some studies revealed that even if the compression on the pituitary stalk was relieved after the resection of a pituitary macroadenoma, the position of EPPHS relative to the pituitary stalk did not change, and the hyperintense signal of the normal posterior pituitary did not reappear. The EPPHS can also show enhancement characteristics similar to a normal posterior pituitary, suggesting that it has a good blood supply (14). These results suggest that the formation of EPPHS in pituitary macroadenoma is accompanied by complex pathophysiological changes. Some animal and human studies have confirmed that after the hypothalamic neurohypophyseal axis is partially severed, the nerve fiber bundle at the proximal end of the injury can proliferate and reconstruct to form an ectopic posterior pituitary tissue. This tissue has abundant blood vessels and a large number of neuroendocrine substances and is functionally equivalent to the normal posterior pituitary (15–17). Therefore, it is speculated that the formation of EPPHS in pituitary macroadenoma may be related to the compression and injury of the hard diaphragm on the pituitary stalk during the growth of pituitary macroadenoma. This injury may cause the reconstruction of the proximal nerve fiber bundles and ectopic posterior pituitary tissue formation and then show a hyperintense signal on T_1_WI.

The formation of ectopic posterior pituitary tissue mainly depends on the injury level of the hypothalamic neurohypophyseal axis. When the injury is located in the hypothalamus, no new ectopic posterior pituitary tissue will be formed (18, 19). A variety of pituitary inflammatory lesions (pituitary granuloma, Langerhans histiocytosis, lymphocytic hypophysitis, etc.) and sellar tumor lesions (craniopharyngioma, germ cell tumor, sellar glioma, lymphoma, metastasis, etc.) often involve the hypothalamus. These diseases are often accompanied by the occurrence of central diabetes insipidus and the disappearance of hyperintense signals in the posterior pituitary (20–22). Six cases (75%) of craniopharyngioma, four cases (100%) of germ cell tumors, one case (100%) of glioma, two cases (100%) of pituitary metastases, and two cases (100%) of pituitary inflammatory diseases did not show hyperintense signals in the posterior pituitary. There was no EPPHS in nine cases of Rathke cleft cyst and 14 cases of tuberculum sellae meningioma. The authors of this study speculate that the former may be due to the soft texture of the lesion, which is not enough to compress and damage the pituitary stalk. The latter mainly compresses the adenohypophysis in the process of growing into the sella, which is not closely related to the spatial position of the neurohypophysis. Therefore, it may not lead to the compression and injury of the pituitary stalk. In addition, some Rathke cleft cysts are accompanied by the disappearance of hyperintense signals in the posterior pituitary. This is considered to be related to the inflammatory response secondary to a Rathke cleft cyst. A previous study revealed the incidence of a hyperintense signal in the posterior pituitary of patients with Rathke cleft cysts with an inflammatory response that was significantly lower than that of patients with Rathke cleft cysts without an inflammatory response (23).

## Conclusion

When sellar tumors or tumor-like lesions are large, they often need to be differentiated from pituitary macroadenoma. Additionally, EPPHS is very common in pituitary macroadenomas. In sellar tumors or tumor-like lesions involving both intrasellar and extrasellar regions, the appearance of EPPHS is helpful to the diagnosis and differential diagnosis of pituitary macroadenoma. The formation of EPPHS in pituitary macroadenoma may be related to the injury of the pituitary stalk.

## Data availability statement

The original contributions presented in the study are included in the article/supplementary material. Further inquiries can be directed to the corresponding author.

## Author contributions

Conception and design of the research, Y-LL, FG, and XL. Acquisition of data, YY and L-KL. Analysis and interpretation of the data, Y-LL, H-QF, and J-HD. Statistical analysis, Y-LL, FG, and XL. Obtaining financing, XL. Writing of the manuscript, Y-LL and FG. Critical revision of the manuscript for intellectual content, XL. All authors contributed to the article and approved the submitted version.

## Funding

This study was funded by the National Natural Science Foundation of China (Grant No. 81960537).

## Acknowledgments

We are particularly grateful to all the people who have given us help on our article.

## Conflict of interest

The authors declare that the research was conducted in the absence of any commercial or financial relationships that could be construed as a potential conflict of interest.

## Publisher’s note

All claims expressed in this article are solely those of the authors and do not necessarily represent those of their affiliated organizations, or those of the publisher, the editors and the reviewers. Any product that may be evaluated in this article, or claim that may be made by its manufacturer, is not guaranteed or endorsed by the publisher.

## References

[1] Zamora C Castillo M. Sellar and parasellar imaging. Neurosurgery (2017) 80(1):17–38. doi: 10.1093/neuros/nyw013 28362892

[2] Li A Liu W Cao P Zheng Y Bu Z Zhou T. Endoscopic versus microscopic transsphenoidal surgery in the treatment of pituitary adenoma: A systematic review and meta-analysis. World Neurosurg (2017) 101:236–46. doi: 10.1016/j.wneu.2017.01.022 28104521

[3] Joshi MN Whitelaw BC Carroll PV. Mechanisms in endocrinology: Hypophysitis: diagnosis and treatment. Eur J Endocrinol (2018) 179(3):R151–63. doi: 10.1530/EJE-17-0009 29880706

[4] Barkhoudarian G Palejwala SK Ansari S Eisenberg AA Huang X Griffiths CF. Rathke's cleft cysts: A 6-year experience of surgery vs. observation with comparative volumetric analysis. Pituitary (2019) 22(4):362–71. doi: 10.1007/s11102-019-00962-y 31016554

[5] Giammattei L Starnoni D Cossu G Bruneau M Cavallo LM Cappabianca P. Surgical management of tuberculum sellae meningiomas: Myths, facts, and controversies. Acta Neurochir (Wien) (2020) 162(3):631–40. doi: 10.1007/s00701-019-04114-w 31834502

[6] Kutin MA Fomichev DV Shkarubo AN Chernov IV Sharipov OI Andreev DN. Endoscopic transsphenoidal approach in treatment of germinomas of the chiasmosellar region. Asian J Neurosurg (2019) 14(4):1190–5. doi: 10.4103/ajns.AJNS_156_19 PMC6896623 31903361

[7] Gao L Guo X Tian R Wang Q Feng M Bao X. Pituitary abscess: clinical manifestations, diagnosis and treatment of 66 cases from a large pituitary center over 23 years. Pituitary (2017) 20(2):189–94. doi: 10.1007/s11102-016-0757-7 27696121

[8] Klyn V Dekeyzer S Van Eetvelde R Roels P Vergauwen O Devolder P. Presence of the posterior pituitary bright spot sign on MRI in the general population: A comparison between 1.5 and 3T MRI and between 2D-T1 spin-echo- and 3D-T1 gradient-echo sequences. Pituitary (2018) 21(4):379–83 doi: 10.1007/s11102-018-0885-3 29594809

[9] Wang CZ Guo LL Han BY Su X Guo QH Mu YM. Pituitary stalk interruption syndrome: From clinical findings to pathogenesis. J Neuroendocrinol (2017) 29(1): 1–7. doi: 10.1111/jne.12451 27917547

[10] Bonneville J-F Bonneville F. The Ectopic Posterior Lobe. In Bonneville JF Bonneville F Cattin F Naggi S editors. MRI of the Pituitary Gland. Cham, Switzerland: Springer (2016). 347–54.

[11] Saeki N Hayasaka M Murai H Kubota M Tatsuno I Takanashi J. Posterior pituitary bright spot in large adenomas: MR assessment of its disappearance or relocation along the stalk. Radiology (2003) 226(2):359–65. doi: 10.1148/radiol.2262011616 12563126

[12] Vilar L Vilar CF Lyra R Freitas MDC. Pitfalls in the diagnostic evaluation of hyperprolactinemia. Neuroendocrinology (2019) 109(1):7–19. doi: 10.1159/000499694 30889571

[13] Eren E Törel Ergür A İşgüven ŞP Çelebi Bitkin E Berberoğlu M Şıklar Z. Clinical and laboratory characteristics of hyperprolactinemia in children and adolescents: National survey. J Clin Res Pediatr Endocrinol (2019) 11(2):149–56. doi: 10.4274/jcrpe.galenos.2018.2018.0206 PMC6571533 30396878

[14] Takahashi T Miki Y Takahashi JA Kanagaki M Yamamoto A Fushimi Y. Ectopic posterior pituitary high signal in preoperative and postoperative macroadenomas: dynamic MR imaging. Eur J Radiol (2005) 55(1):84–91. doi: 10.1016/j.ejrad.2004.10.003 15950103

[15] Daniel PM Prichard MM. Regeneration of hypothalamic nerve fibres after hypophysectomy in the goat. Acta Endocrinol (Copenh) (1970) 64(4):696–704. doi: 10.1530/acta.0.0640696 5468667

[16] Antunes JL Louis KM Huang S Zimmerman E Carmel PW Ferin M. Section of the pituitary stalk in the rhesus monkey: Morphological and endocrine observations. Ann Neurol (1980) 8(3):308–16. doi: 10.1002/ana.410080315 7002018

[17] Daniel PM Prichard MM. The human hypothalamus and pituitary stalk after hypophysectomy or pituitary stalk section. Brain (1972) 95(4):813–24. doi: 10.1093/brain/95.4.813 4647153

[18] Dellmann HD. Degeneration and regeneration of neurosecretory systems. Int Rev Cytol (1973) 36:215–315. doi: 10.1016/S0074-7696(08)60219-3 4587389

[19] Ruszała A Wójcik M Krystynowicz A Starzyk J. Distinguishing between post-trauma pituitary stalk disruption and genetic pituitary stalk interruption syndrome - case presentation and literature overview. Pediatr Endocrinol Diabetes Metab (2019) 25(3):155–62. doi: 10.5114/pedm.2019.87708. 31769274

[20] Patti G Ibba A Morana G Napoli F Fava D di Iorgi N. Central diabetes insipidus in children: Diagnosis and management. Best Pract Res Clin Endocrinol Metab (2020) 34(5):101440. doi: 10.1016/j.beem.2020.101440 32646670

[21] Shah LM Phillips CD. Imaging sellar and suprasellar pathology. Appl Radiol (2009) 38(4):9–21. doi: 10.37549/AR1686

[22] Caranci F Leone G Ponsiglione A Muto M Tortora F Muto M. Imaging findings in hypophysitis: A review. Radiol Med (2020) 125(3):319–28. doi: 10.1007/s11547-019-01120-x 31863360

[23] Wang S Nie Q Wu Z Zhang J Wei L. MRI And pathological features of rathke cleft cysts in the sellar region. Exp Ther Med (2020) 19(1):611–8 doi: 10.3892/etm.2019.8272 PMC6923755 31897104

